# Effect of floor versus conventional elevated housing on the welfareand meat quality of rabbits

**DOI:** 10.5455/javar.2025.l984

**Published:** 2025-12-25

**Authors:** José Manuel Robles-Robles, Adrián Muñoz-Cuautle, José Luis Ponce-Covarrubias, María Esther Ortega-Cerrilla, Fernando Utrera-Quintana, Ricardo Martínez-Martínez, Abel Villa-Mancera

**Affiliations:** 1 Facultad de Medicina Veterinaria y Zootecnia, Benemérita Universidad Autónoma de Puebla, Tecamachalco Puebla, México; 2 Universidad Autónoma de Guerrero, Escuela Superior de Medicina Veterinaria y Zootecnia No.3, Tecpán de Galeana, México; 3 Colegio de Postgraduados Campus Montecillo, Texcoco, México; 4 Centro Universitario de la Costa Sur de la Universidad Autónoma de Guadalajara, Jalisco, México

**Keywords:** Floor housing, Floor hutch, Meat quality, Welfare, Carcass, Rabbit housing

## Abstract

**Objective:**

The housing system is a crucial determinant of animal welfare. This study aimed to compare the effects of intensive housing with those of alternative floor housing on the welfare of rabbits and their meat production.

**Materials and Methods:**

Eighty New Zealand rabbits (40 females and 40 males) with an initial mean weight of 1.3 kg and aged 35 days were distributed into four treatment groups. Treatment 1 (T1) comprised five floor cages with four male rabbits in each cage; Treatment 2 (T2) comprised five floor cages, each containing four female rabbits; Treatment 3 (T3) consisted of five elevated cages housing four male rabbits each, and Treatment 4 (T4) consisted of five elevated cages housing four female rabbits each. Indicators of carcass quality, including moisture, protein, lipids, ash, pH, color (L*, a*, b*), physiological stress (plasma cortisol), oxidative stress (lipid oxidation TBARS), and number of antioxidants (FRAP) in the plasma and tissue, were measured.

**Results:**

A significant difference (*p* < 0.05) in pH was observed between the conventional cages and the floor cages, as well as in cortisol levels for the traditional and floor cages (*p* < 0.05), respectively. Additionally, lipid oxidation (TBARS) was not significantly different in plasma, but was significantly different in meat. The TBARS value was higher for floor cages and lower for conventional cages. The FRAP value was not significantly different between plasma and meat (*p* < 0.05). The findings demonstrate that the use of floor cages does not affect the nutritive value of rabbit meat.

**Conclusion:**

The type of cage used affects rabbits’ physiological stress levels and lipid oxidation in muscle tissue, which impacts meat quality. The nutritional value of meat remains unaffected, regardless of the cage type or sex of the rabbit. The floor cage environment enables the rabbits to engage in activities typical of their species, thereby contributing to the animals’ welfare by improving driving skills, attitudes, and handler behavior.

## Introduction

Animal welfare is currently a significant factor in livestock production. Regarding rabbits bred for meat, consumers have shown increasing demand for welfare-friendly rabbit farming practices. Adequate housing is critical for animal welfare [1-3]. In Mexico, traditional pen cages are used for housing rabbits. There has been very little research on the use of floor hutches in rabbit production, despite the observation that floor cages promote species-specific behaviors and contribute to the animals’ welfare. In Egypt, rabbits weaned at 35 days of age and reared in cages exhibited higher carcass traits and meat quality compared to those weaned at 28 days and reared in floor houses [4]. The floor housing allows the rabbits to exhibit their innate behaviors, which are typically observed in the wild. However, it has been demonstrated that rabbits housed in cages often exhibit aggressive behavior, yet they also display social tendencies, such as hopping and interacting with their cage mates [5]. Research has focused on nutrition, genetics, and reproduction, leaving aside the issue of animal welfare in livestock farming [6]. The effects of housing type on rabbit behavior have been demonstrated in previous studies. Housing rabbits in pen cages with elevated platforms increases footfall between animals compared to pen housing. Open pens allow for alert behaviors that are not expressed in cages with low ceiling heights [7]. The concentration of cortisol in the plasma is higher in rabbits housed in groups compared to those housed individually, with a lower cortisol concentration resulting in less physical damage [1]. Housing in dense groups presents a greater degree of physical damage [8]. Szendrő and Zotte [9] have demonstrated through numerous experiments involving the growth of rabbits that higher group sizes result in greater stress levels, lower feed intake and weight gain, decreased slaughter performance, increased infection and mortality rates, and a higher incidence of lesions caused by aggression.

Stress is influenced by the type of housing for rabbits, and stress affects the secretion of cortisol. Cortisol is a hormone that enables the animal to react to emergencies [10], including the mobilization of muscle and liver glycogen reserves. These energy-obtaining processes can acidify the meat [11]. Cortisol is also related to the fat content of meat, as has been reported in pigs [12] and in beef cattle [10]. Cortisol, when measured directly in the animal, can be an indicator of stress [13]. An additional effect of housing type is lipid oxidation [14], which is caused by the presence of reactive oxygen species. Lipid oxidation can impact the shelf life and physicochemical properties of meat. The above discussion suggests that the type of housing can have implications for the final product of rabbit husbandry and that the effect can be measured by the concentration of cortisol [15], the level of lipid oxidation, TBARS, and the concentrations of antioxidants (FRAP) [16]. Therefore, the aim of this study was to investigate the impact of two types of floor housing on the welfare and meat quality of rabbits.

## Materials and Methods

### Ethical approval

The research was approved by the Ethics and Animal Care Committee of the Benemeritous Autonomous University of Puebla, and all procedures complied with the National Legislation on Animal Health Research (458742).

### Study area

This study was carried out at the Animal Husbandry Station of “El Salado,” associated with the Faculty of Veterinary Medicine and Zootechnics from Benemeritus Autonomous University of Puebla, Mexico (18°52’ N and 97°43’ W). The study area has an elevation of 2055 m above sea level, characterized by a semi-arid temperate climate with summer precipitation, and an average annual rainfall and temperature of 700 mm and 18°C, respectively [17].

### Experimental units

The experiment was conducted over 40 days, spanning February to March 2024. The rabbits were slaughtered and sampled at 75 days of age, demonstrating a final average weight of 2.07 ± 0.05 kg. A total of 40 female and 40 male New Zealand rabbits aged 35 ± 7 days with an initial average weight of 1.324 kg were distributed among four treatments. In Treatment 1 (T1), five floor cages were designed to accommodate four male rabbits each, and in Treatment 2 (T2), five floor cages were used to hold five female rabbits each; Treatment 3 (T3) comprised five elevated cages designed to accommodate four male rabbits each, and in Treatment 4 (T4), five elevated cages were used to hold four female rabbits each. The elevated cages had a surface area of 0.45 m² per animal. The floor housing measured 1.2 m in width, 1.5 m in length, and 0.5 m in height. The cages were lined with galvanized wire mesh, with openings measuring 4.5 cm at the top and 2.5 cm at the bottom. The floor of each cage was also lined with mesh, with openings measuring 2.5 cm. This was deemed sufficient to ensure the physical comfort of the rabbits [18]. The nest boxes were constructed from wood and measured 30 cm in width, 40 cm in height, and 60 cm in length, with a circular entrance at the front measuring 20 cm in diameter. They also featured hopper feeders and automatic waterers. The second housing system was conventional or elevated cages, comprising ten wire cages measuring 90 cm in length, 60 cm in width, and 40 cm in height. These cages were elevated one meter from the ground level and were constructed with a metal frame. The cages were equipped with a water system comprising troughs and a hopper feeder. The density of the population was 0.135 m² per animal, with four rabbits per cage.

### Feeding

The rabbits were given food and water *ad libitum* and were fed a commercial diet formulated specifically for rabbits. The nutritional composition of the commercial feed was previously reported by Robles et al. [19].

### Statistical analysis

The data were examined utilizing a completely randomized design with a 2 × 2 factorial arrangement. The model for analysis included the main effects of cage (conventional and floor) and sex (females and males), and their interactions. Five replicates were used per treatment combination, with four rabbits per replicate. The GLM procedure of SAS (SAS, 2010) was utilized for analysis, and the means were compared using the Tukey test. Data were expressed as means ± S.E.

The data were examined using the following model:

*Y*_*ijk*_ = μ + *A*_*i*_ +*B*_*j*_ + *(AB)*_*ij*_+*ε*_*ijk*_

where *Y*_*ijk*_ = the effects of humidity, protein, lipids, ash, pH, color, cortisol in serum, lipid oxidation in plasma and muscle tissue, and the presence of antioxidants in plasma and muscle tissue.

 μ = the overall (grand) Mean

* A*_*i*_ = the effect of the *i*^*th*^ type of cage (1,2).

* B*_*j*_ = effect of the *j*^*th*^ sex (1,2).

*AB*_*ij*_ = Cage × Sex interaction.

* ε* = Error term.

## Results

The interaction between the different factors was not significant (*p* > 0.05); therefore, only the main effects are discussed. The results for cortisol concentrations (Table 1) showed a significant difference between treatments (*p* < 0.05). In the pen (elevated) cages, the cortisol concentration was undetectable, while in the floor hutches, the concentration was higher (0.11 pg/ml). The oxidation of lipids in the blood plasma of rabbits was measured using the TBARS technique, and the antioxidant concentrations were determined by the FRAP method. No significant differences were observed (*p* > 0.05) between cages or between sexes (Table 2).

**Table 1.** Cortisol, lipid oxidation and antioxidants present in serum and blood plasma of rabbits housed in floor cages (T1 and T2) and in conventional (elevated) cages (T3 and T4).

**Treatment/housing type/sex**	***n***	**Cortisol (pg/ml) (± S.E)**	**TBARS (nmol/100µl) (± S.E)**	**FRAP (nmol/50µl) % (± S.E)**
T1/male/floor cage	20	0.07 ±0.05^a^	2.46 ± 1.31	15.15 ± 4.01
T2/ female/floor cage	20	0.11 ± 0.17^a^	2.89 ± 0.53	12.45 ± 2.28
T3/male/elevated cage	20	–	2.31 ± 0.83	13.84 ± 3.06
T4/female/elevated cage	20	–	3.71 ± 1.06	15.99 ± 7.61

S.E., standard error of the mean. ^a, b^ Different letters in the same row indicate differences (*p* < 0.05).

**Table 2.** Effect of treatments on lipid oxidation and antioxidant concentration in meat from fattening rabbits.

**Treatment/housing type/sex**	***n***	**TBARS (nmol/ 100 µl) (± S.E)**	**FRAP (nmol/** **50 µl)** ** % (± S.E)**
T1/male/floor cage	20	15.74 ± 1.66^a^	21.64 ± 7.15
T2/ female/floor cage	20	17.21 ± 2.07^a^	19.95 ± 7.26
T3/male/elevated cage	20	13.60 ± 3.31^b^	29.33 ± 9.92
T4/female/elevated cage	20	13.53 ± 4.33^b^	27.81 ± 12.16

S.E., standard error of the mean. ^a, b^ Different letters in the same row indicate differences (*p* < 0.05).

There were no significant differences (*p* > 0.05) in humidity, protein, ether extract, or ash content due to the treatments. The pH levels differed between cage types, with higher values in the conventional cages and lower values in the floor cages. The light and color associated with the treatments were not statistically significant (*p* > 0.05) (Table 3).

**Table 3.** Meat characteristics of finishing New Zealand rabbits according to cage type, sex and their interaction

**Treatment/Housing type/sex**	***n***	**Humidity % (± S.E)**	**Proteins %** **(± S.E)**	**Ether extract % (± S.E)**	**Ashes%** **(± S.E)**	**pH (± S.E)**	**Color**		
							L*(± S.E)	a*(±S.E)	b*(± S.E)
T1/male/floor cage	20	98.18 ± 0.64	20.07 ± 1.27	6.11 ± 1.14	1.85 ± 0.66	5.76 ± 0.04^a^	55.47 ± 3.65	12.94 ± 2.02	5.47 ± 1.93
T2/ female/floor cage	20	98.53 ± 0.25	19.55 ± 1.65	6.59 ± 1.33	1.48 ± 0.26	5.78 ± 0.09^a^	58.77 ± 2.194	11.80 ± 1.68	4.69 ± 1.92
T3/male/elevated cage	20	98.36 ± 0.56	20.02 ± 0.91	5.72 ± 0.82	1.66 ± 0.58	5.82 ± 0.04^b^	60.11 ± 1.182	12.00 ± 4.06	5.38 ± 2.63
T4/female/elevated cage	20	98.79 ± 0.19	20.78 ± 0.86	6.69 ± 0.55	1.21 ± 0.20	5.84 ± 0.04^b^	57.48 ± 2.02	12.83 ± 1.01	5.19 ± 0.56

L*= Luminosity; a*= Coloration from red to green; b*= Coloring from yellow to blue. S.E., standard error of the mean.

^a,b^; Different letters in the same row indicate differences (*p* < 0.05).

## Discussion

The production of fattening rabbits can be affected by multiple factors, one of which is the type of housing, which is based on the system’s specifications and includes the types of materials used in the space allocated for specific behaviors, such as movement, rest, and feeding. It also includes other unique rearing settings that the system offers, such as health and/or social stress [2,20,21].

Some authors have shown that conventional pen cages, such as those used in this study, limit the opportunity for social interaction; with less space, rabbits exhibit lower cortisol concentrations [22]. Elevated cortisol levels in rabbits kept in floor hutches may be due to the greater amount of space, as this allows for fighting to establish dominance hierarchies and greater mobility, activities typical of the species. Apparently, rabbits require a specific concentration of cortisol to maintain alertness and ensure survival. This alertness system is manifested in floor hutches. Hube et al. [15] found that cortisol concentration increased in groups of three rabbits compared to those kept individually. Bozzo et al. [22] observed similar results when comparing open cages vs. conventional pen cages and industrial systems.

The FRAP in the plasma reflects antioxidant activity determined by the reduction of ferric ions [23]. High magnitudes of this indicator are associated with the integrity of the organism’s cell membranes; lipid compounds are crucial for maintaining cell health. In our study, there was no difference in FRAP associated with the type of housing. This suggests that housing did not affect antioxidant activity. The type of diet has been shown to be a source of variation in antioxidant activity. Ebeid et al. [24] supplemented rabbits’ diets with vitamin E and observed enhanced antioxidant activity. However, Mattioli et al.[25] found no difference in plasma FRAP in rabbits fed with olive leaves enriched with selenium.

TBARS is an indicator of the degree of oxidative stress within a biological sample [26]. No differences were observed in plasma TBARS between the types of housing or sexes in the rabbits; this may have been due to the absence of additional stressors or to the physical activity of the rabbits. The results of the evaluation of rabbit meat quality characteristics evaluated in this study (moisture, protein, ether extract, and ash) are similar to those reported by Dalle-Zotte et al. [27]. In the latter study, which examined different types of housing, no differences were observed in meat quality.

Sampels and Skoglund [28] reported a variation attributable to sex, with higher values in females (6.0) and lower values in males (5.8), a result attributed to less tension in females. The properties of meat, particularly its color parameters, are closely associated with the pH level [21]. This study found no differences in pH associated with the sex of the rabbits; however, differences were observed depending on the type of housing. Rabbits housed in conventional pen cages had a higher pH value than those housed in floor hutches. The decreased pH in floor hutches (T1 and T2) was associated with greater physical activity, which produced higher catabolism of glycogen into lactic acid, causing a decrease in the pH of the meat.

Paci et al. [29] reported that rabbits housed in conventional pen cages at a low density had a lower pH than those housed in pen cages at a high density. With limited space, the rabbits displayed aggressive behaviors, and the stress affected the final pH of the meat. The pH can impact the quality of the meat, as it affects the water retention capacity. However, the pH values obtained in this study are adequate for maintaining the normal shelf life of rabbit meat, as indicated by Menchetti et al. [30].

The color of meat is one of the most prominent attributes for consumers and is evaluated initially. The outcome is attributable to qualitative alterations in the composition of meat, mostly due to modifications in myoglobin and hemoglobin [31]. The meat color parameters L*, a*, and b* were unaffected by the increased physical activity associated with floor housing. Differences in meat color have been reported due to the type of diet [32], breed [33], the age of the animal [34], the time of measurement [28], and even the instrument with which the measurement is carried out [35]. In contrast to the values obtained in this study, where the values of L*, a*, and b* were not significantly different (*p* > 0.05), Krunt et al. [21] observed differences in the color variable a* when evaluating rabbit housing in pens (less red color) and in conventional pen cages (more red color). Krunt et al. [21] examined the longissimus thoracis muscle, while this study evaluated the longissimus dorsi muscle. Due to the greater activity of the thoracic muscle, the greater redness is reasonable compared to the longissimus dorsi muscle, which experiences less physical effort.

The oxidant activity (TBARS) in rabbit meat differed between housing treatments, being higher for rabbits housed in floor hutches compared to conventional pen cages. The greater space in the floor hutch allows for greater movement, and this, in turn, increases the generation of free radicals and may enhance oxidative activity [14]. The TBARS recorded values were higher in rabbits housed in floor hutches, indicating higher levels of cortisol, a result attributed to enhanced physical activity and greater space in which to manifest alert behaviors.

## Conclusion

The present study has demonstrated that the type of cage affects the physiological stress level of rabbits, as well as the lipid oxidation of the muscle tissue, and hence, the quality of the meat. The nutritional value of the meat was unaffected by the type of cage or the sex of the rabbits. Floor cages represent an alternative housing system in commercial rabbit farming, as they do not affect the nutritional quality of the meat, and they contribute to the animals’ well-being, allowing them to engage in typical species-specific behaviors.

## List of abbreviations

a, Coloration from red to green; b, Coloring from yellow to blue; cm, centimeters; FRAP, ferric reducing antioxidant power; kg, kilogram; L, Luminosity; m, meters; m², square meters; pH, hydrogen potential; S.E., standard error; TBARS, thiobarbituric acid reactive substances; T, Treatment.
